# Upper extremity musculoskeletal disorders and exposure to Ergonomic risk factors among handicraft workers

**DOI:** 10.12669/pjms.37.2.749

**Published:** 2021

**Authors:** Wajeeha Mahmood, Muhammad Salman Bashir, Sarah Ehsan, Muhammad Atif Qureshi

**Affiliations:** 1 Dr. Wajeeha Mahmood, (MS-NMPT), Senior Lecturer, Department of Physical Therapy, Superior University, Lahore, Pakistan; 2 Prof. Dr. Muhammad Salman Bashir (PhD-PT) Riphah College of Rehabilitation & Allied Health Sciences, Lahore. Faculty of Rehabilitation and Allied Health Sciences, Riphah International University, Islamabad, Pakistan; 3 Dr. Sarah Ehsan, (PP-DPT) Senior Lecturer, Faculty of Rehabilitation and Allied Health Sciences, Riphah International University, Islamabad; 4 Prof. Dr. Muhammad Atif Qureshi, Department of Medicine, Azra Naheed Medical College, Lahore, Pakistan

**Keywords:** Ergonomics, Musculoskeletal diseases, Pain, Risk factors, Shoulder, Upper Extremity

## Abstract

**Objective:**

To determine the association of Upper extremity musculoskeletal disorders and Exposure to Ergonomic risk factors among handicraft workers.

**Methods:**

This cross-sectional survey was conducted in a 12 weeks’ duration i.e. from November 2018 till January 2019. Nordic musculoskeletal questionnaire was used to determine the frequency of upper limb musculoskeletal disorders. Postural analysis was done using Rapid Upper Limb Assessment (RULA). Data was collected from 100 Handicraft workers and the type of work included Art work (n=18), Ada Work (n=7), botanical arrangement (n=11), Textile, fashion designing and stitching (n=38), Fine arts (n=20), embroidery and knitting (n=6).

**Results:**

The frequency distribution of Rapid upper limb assessment (RULA) score for exposure to risk factors showed that 47 participants were at a high risk and required implementation of change. Most of the participants (n=35) reported pain in neck and shoulder (n=29). The chi square test for association between pain in upper extremity and exposure to risk factors showed that only wrist pain had statistically significant association with overall RULA score (p-value<0.05).

**Conclusion:**

The study concluded that neck and shoulder are more frequently affected among handicraft workers. The frequency of workers who were at high ergonomic risk and required implementation of change in working conditions was high.

## INTRODUCTION

The industrial safety is an important concern for the productivity of any small- or large-scale industry. The small-scale industries that involve intensive manual labor and long working hours in awkward postures put the workers at a risk of developing health related issues. There is a large gap between knowledge and actual practice of proper ergonomics in Pakistan. Most of the employees do not follow ergonomic principles at workplace because there is lack of formal training on workplace ergonomics and the risk of developing musculoskeletal disorders.^1^

In Indo-Pak subcontinent, a large number of informal sector workers are involved in small scale industries either in trades or manufacturing. These small scale industries primarily include the handicrafts, village and khadi industries. Handicrafts are the items that are produced by worker’s manual skill and have nuances of the traditions of the cultural values of a society.^2^

Handicraft industry is the main source of livelihood for some communities and has a significant role in determining the socio-economic state of a society.^2^ Handicraft sector is a source of cultural and ecotourism which provides work opportunities to a lot of people.^3^

According to a systematic review handicraft workers are at a high risk of developing musculoskeletal disorders. Work postures, working hours, repetitive tasks, work experience, gender, age and stressful work environment have been identified as the risk factors for work related musculoskeletal disorders (WRMSDs) among handicraft workers. However, it is highlighted that work related risk factors should be explored for identifying the cause of musculoskeletal disorders among handicraft workers.^4^

There is a lack of literature on workplace ergonomics of handicraft workers in Pakistan. The occupational health and safety (OHS) is inadequate in Pakistan owing to reasons like illiteracy. There also is no system of recording occupational and work-related injuries in Pakistan.^5^

Assessment of the ergonomic conditions of other small-scale industries revealed that most of the workers have to work for prolonged durations with a single break in the middle of the day. The factories have poor infra structure and because of the lack of formal education among the workers there is less awareness regarding the proper working postures. The relationship of musculoskeletal disorders with working conditions is still unclear as the psychosocial factors might also have an influence in increasing the risk of work-related musculoskeletal disorders. According to a study conducted on one of the largest garment factories of Pakistan proper attention is not paid to health-related issues at workplace.^6^

It has been reported that most of the small-scale industry workers have to work with traditionally designed tools for the manual work. Repetitive and improper use of these tools poses risk for the development of work-related musculoskeletal disorders. the knowledge and application of proper workplace ergonomics will lead to an increased worker safety, productivity and will also improve revenue generation.^7^

This study hypothesized that Upper extremity musculoskeletal disorders among handicraft workers have significant association with Exposure to Ergonomic risk factors. The objective of this study was to determine the association of Upper extremity musculoskeletal disorders and Exposure to Ergonomic risk factors among handicraft workers.

## METHODS

This cross-sectional survey was conducted in Qasr e Behbood Institute, Lahore within 12 weeks’ duration i.e., from November 2018 till January 2019. The study was approved by the Ethical Review Committee (Ref.No. RCR & AHS/PP-DPT/001 dated November 1, 2018) of the Riphah International University. Workers involved in various types of handicrafts manufacturing tasks were included in the study. The types of Handicrafts included Art work, Ada Work, botanical arrangement, Textile, fashion designing and stitching, Fine arts, embroidery and knitting. A sample of 100 workers was selected using simple random sampling technique. Female handicraft workers with a minimum of one-year experience were included in the study. The minimum criteria of working hours for inclusion in the study was at least five hours per day. Workers were excluded from the study if they had some systemic disease-causing musculoskeletal problem, musculoskeletal injury other than work related like whiplash injury and car accidents or with a history of musculoskeletal surgery.

The data was collected using questionnaires as well as with direct observation of the participants on workstations. The questionnaire consisted of two parts. The first part consisted of questions related to socio demographic characteristics and different work-related factors. The second part consisted of Nordic musculoskeletal questionnaire which was used to determine the frequency of upper limb musculoskeletal disorders. Postural analysis was done using Rapid Upper Limb Assessment (RULA).

Rapid Upper Limb Assessment (RULA) is used to assess postural loading and biomechanical factors acting on complete body with emphasis on upper limb, neck and trunk. RULA is a helpful tool in screening the ergonomic risks at workplace as the final scores of the scale also interpret as to whether an implementation of change is required or not.^6^ The posture analysis using The Rapid upper limb assessment (RULA) is considered a pen-paper observation method.

Workers were observed from a sagittal view and the postures maintained for the longest duration were considered for analysis. The angles were measured using goniometer. Postures of upper arm, lower arm, forearm and wrist were scored and labelled as Posture Score A. Static and repetitive loading; force or load scores were then calculated. Same procedure was repeated for neck, back and legs posture and was labelled as Posture Score B. All the scores were then added and the final scores determined the ergonomic risk of associated with the posture. RULA is a reliable tool and has shown high Interrater reliability.^7^

### Statistical analysis

Statistical analysis was done using SPSS version 20. Frequency and percentage were used for qualitative variables and descriptive statistics were used for quantitative variables. Chi-square test was used to determine the association of Upper extremity musculoskeletal disorders and Exposure to Ergonomic risk factors. P-value of 0.05 was considered significant.

## RESULTS

The mean age of handicraft workers was 25.99±7.97 years with minimum recorded age of 16 years and maximum age of 50 years. The mean weight of the participants was 57.43±11.77 kg and mean height of handicraft workers was 5.29±0.21 feet. The average Body mass Index (BMI) of handicraft workers was 21.88±4.19. The minimum BMI was 14.05 and maximum was 34.80. The frequency of gravid ranged from 0 to 11 times and frequency of parity ranged from Zero to Five times. The mean working hours of handicraft workers were recorded as 5.24±2.57 hours per day. The average resting period of handicraft workers was recorded as 0.36±0.37 hour per day.

Working hours were reported to be between 3 to 6 hours by 69 respondents and more than six hours by 25 respondents. Most of the participants (n=45) reported half hour rest period during work time. The frequency distribution of Rapid upper limb assessment (RULA) score for exposure to risk factors showed that 47 participants were at a high risk and required implementation of change.

**Table-I:** Frequency distribution of Exposure to ergonomic risk factors in Handicraft workers.

	*Frequency (n=100)*
Negligible Risk, No action required	1
Low risk, change may be needed	9
Medium risk, change soon, further investigation	43
Very high risk, implement change now	47

The results from Nordic questionnaire revealed that most of the participants (n=35) reported pain in neck followed by pain in shoulder (n=29). The chi-square test of association showed that the association of type of work was statistically significant with Neck and Elbow pain (p-value <0.05).

The association of Rapid upper limb assessment scale’s individual items showed that neck and wrist pain had statistically significant association with Neck position (p-value<0.05). The trunk position and pain in upper back also showed statistically significant association (p-value< 0.05).

The chi square test for association between pain in upper extremity and exposure to risk factors showed that only wrist pain had statistically significant association with overall RULA score i.e. exposure to ergonomic risk factors (p-value<0.05). No other region showed statistically significant association with overall RULA score (p-value>0.05).

## DISCUSSION

Main purpose of this study was to identify the presence of WRMSDs and to determine their association with ergonomic risk factors in handicraft workers. This identification is necessary to reduce absenteeism of workers and increase productivity of industry. By identification of poor ergonomics, proper posture related interventions could be devised which will reduce WRMSDs in future according to RULA assessment sheet. Handicrafts Industry is an important pillar in a country’s economy therefore, it is very important to assess the work related musculoskeletal disorders among handicraft workers.

**Table-II:** Association of ergonomic risk factors with Upper extremity musculoskeletal disorders in handicraft workers

*Ergonomic risk (n=100)*						

*Negligible*		*Low*	*Medium*	*Very high*		*P-value*
Neck	Yes	-	-	18	17	0.09
	No	1	9	25	30	
Shoulder	Yes	-	1	18	10	0.07
	No	1	8	25	37	
Elbow	Yes	-	-	-	2	0.89
	No	1	9	43	45	
Wrist	Yes	-	3	3	2	0.00
	No	1	6	40	45	
Upper Back	Yes	-	-	3	-	0.25
	No	1	9	40	47	

**Table-III:** Exposure to ergonomic risk and the work-related factors in handicraft workers.

				*Ergonomic risk*		

		*Negligible*	*Low*	*Medium*	*Very high*	*P-value*
Type of Work	Art Work	-	2	9	7	0.00
	Adda Work	-	-	5	2	
	Botanical arrangement	-	2	5	4	
	Textile, fashion designing and stitching	1	1	6	30	
	Fine arts	-	4	13	3	
	Embroidery and knitting	-	-	5	1	
Working hours	Less than 3	-	2	1	3	0.22
	3 to 6	1	7	29	32	
	More than 6	-	-	13	12	
Working position	Standing	-	2	-	7	0.18
	Sitting with support	-	1	3	3	
	Sitting without support	1	6	40	37	

The results showed that pain in neck and shoulder was prevalent among the workers. Our findings suggest that wrist pain has statistically significant association with ergonomic risk factors. This shows that among handicraft workers wrists are most prone to developing pain because of poor ergonomics. The RULA assessment showed that most of the participants required implementation of change at workplace as they were at high risk of developing musculoskeletal problems. The study hypothesis was accepted as there was a significant association between the ergonomic risk and upper extremity musculoskeletal problems.

The results of current study are supported by previous literature. Garment factory workers have reported musculoskeletal problems associated with shoulders, upper and lower back. It was reported that their workstations had poor ergonomic design and required some major changes. These results support our findings as the working postures in garment factories and handicraft workers are relatable.^7^

Results of a Literature review has shown that application of ergonomic principles was more evident in large scale industries rather and small scale industries still lag behind despite of the fact that small scale industries require more manual labor.^8^ Similar results have been reported by Shakerian et al. (2016)^9^ in Iranian handicraft workers. They studied the impact of work place physical activity on musculoskeletal discomfort among handicraft workers. Pain in shoulder, wrist, neck and upper arm were prevalent and physical activity has statistically significant association with Musculoskeletal discomfort in wrist, low back and neck (p-value <0.05).

Singh^10^ conducted a study on female workers of chikankari industry in Lucknow, India. REBA was used for postural analysis. It was concluded that ergonomic principles must be implemented in order to prevent musculoskeletal problems in Chikankari industry.It has been suggested that suitable ergonomic interventions should be applied in order to ensure workers’ productivity and risk prevention.^11^ A study conducted on Iranian shoe-sole makers has shown that musculoskeletal problems were significantly associated with ergonomic factors.^12^

A review by Mrunalini and Logeswari (2016)^13^ in India showed that musculoskeletal disorders were prevalent among handicraft workers. they reported that less literature was available on the association of musculoskeletal disorders and working postures in handicraft workers. Few studies were found regarding the preventive strategies for musculoskeletal disorders in handicraft workers.

Sain and Meena (2018) explored the musculoskeletal problems and risk factors associated with them in brick kiln workers. Rapid Upper limb assessment scale (RULA) and Rapid entire body assessment (REBA) were used for posture analysis and exposure to risk factors. It was reported that the RULA scores for spading, mold filling, mold evacuating and brick carrying were high. The results are consistent with our results.^14^

As handicraft industry is an integral part of the industrial system of Pakistan, the workplace safety and productivity of the artisans is very important. Musculoskeletal problems limit the work capacity of handicraft workers and therefore creates a hindrance in their work. In order to ensure productivity and workers’ well-being further studies should be conducted to explore the factors that are creating a lapse in the application of ergonomic principles in the handicrafts industry. Moreover, it is recommended that the future research should also focus on determining the effectiveness of various ergonomic strategies in reducing the risk and frequency of work-related musculoskeletal disorders among handicraft workers.

## CONCLUSION

The study concluded that neck and shoulder are more frequently affected among handicraft workers. The frequency of workers who were at high ergonomic risk and required implementation of change in working conditions was high. Only pain in the wrist showed significant association with ergonomic risk factors i.e., overall RULA score. Type of work also had significant association with ergonomic risk factors with most of the workers in Textile, fashion designing and stitching falling in the high-risk category requiring implementation of change.

### Authors’ Contribution:

**WM, MSB and SE:** Conceived, designed and did statistical analysis & editing of manuscript.

**WM, MSB, SE and MAQ:** Did data collection and manuscript writing.

**SE and MAQ:** Critical review, final approval of manuscript and are responsible for integrity of the study.
